# Diacetatodi-*tert*-butyltin(IV)

**DOI:** 10.1107/S1600536813009185

**Published:** 2013-04-13

**Authors:** Martin Reichelt, Hans Reuter

**Affiliations:** a Institut für Chemie neuer Materialien, Strukturchemie, Universität Osnabrück, Barbarastr. 7, D-49069 Osnabrück, Germany

## Abstract

The title compound, [Sn(C_4_H_9_)_2_(CH_3_COO)_2_], was synthesized in order to study the influence of large organic groups on the mol­ecular structure of diorganotin di­acetates. The title compound exhibits the same structure type as other diorganotin(IV) di­acetates characterized by an unsymmetrical bidentate bonding mode of the two acetate groups to tin. The influence of the *t*-butyl groups on this mol­ecular structure is expressed in two significant differences: tin—carbon bond lengths are much more longer than in the other di­acetates, as are the additional inter­actions of the acetate groups with the tin atom. Inter­molecular inter­actions are restricted to C—H⋯O ones similar to those in the other di­acetates, giving rise to a chain-like arrangement of the molecules with the tin atoms and acetate groups in the propagation plane.

## Related literature

For background to diorganotin(IV) carboxyl­ates, see: Tiekink (1991 ▶) and to diorganotin(IV) di­acetates, see: Alcock *et al.* (1992 ▶); Lockhart *et al.* (1987 ▶); Mistry *et al.* (1995 ▶).
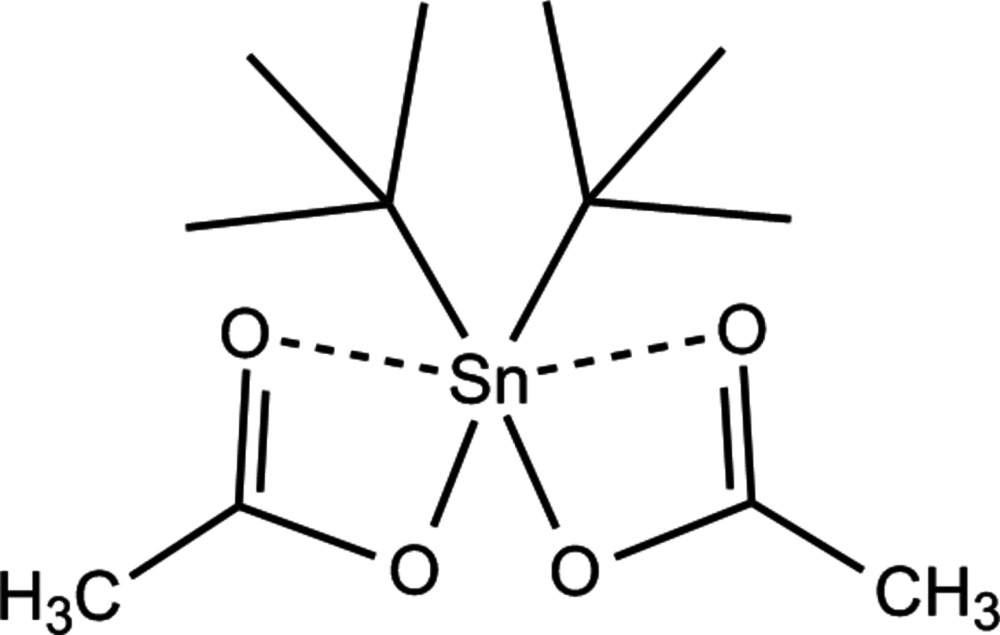


## Experimental

### 

#### Crystal data

[Sn(C_4_H_9_)_2_(C_2_H_3_O_2_)_2_]
*M* *_r_* = 351.00
Monoclinic, 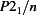

*a* = 6.1039 (3) Å
*b* = 15.3928 (7) Å
*c* = 15.9601 (8) Å
β = 95.462 (2)°
*V* = 1492.74 (12) Å^3^
*Z* = 4
Mo *K*α radiation
μ = 1.71 mm^−1^
*T* = 100 K
0.14 × 0.06 × 0.04 mm

#### Data collection

Bruker APEXII CCD diffractometer
Absorption correction: multi-scan (*SADABS*; Bruker, 2009 ▶) *T*_min_ = 0.791, *T*_max_ = 0.939
76636 measured reflections
3590 independent reflections
2980 reflections with *I* > 2σ(*I*)
*R*_int_ = 0.081

#### Refinement

*R*[*F* ^2^ > 2σ(*F* ^2^)] = 0.023
*wR*(*F* ^2^) = 0.046
*S* = 1.04
3590 reflections
167 parameters
H-atom parameters constrained
Δρ_max_ = 0.59 e Å^−3^
Δρ_min_ = −0.51 e Å^−3^

### 

Data collection: *APEX2* (Bruker, 2009 ▶); cell refinement: *SAINT* (Bruker, 2009 ▶); data reduction: *SAINT*; program(s) used to solve structure: *SHELXS97* (Sheldrick, 2008 ▶); program(s) used to refine structure: *SHELXL97* (Sheldrick, 2008 ▶); molecular graphics: *DIAMOND* (Brandenburg, 2006 ▶) and *Mercury* (Macrae *et al.*, 2008 ▶); software used to prepare material for publication: *SHELXTL* (Sheldrick, 2008 ▶).

## Supplementary Material

Crystal structure: contains datablock(s) I, global. DOI: 10.1107/S1600536813009185/aa2086sup1.cif

Structure factors: contains datablock(s) I. DOI: 10.1107/S1600536813009185/aa2086Isup2.hkl

Additional supplementary materials:  crystallographic information; 3D view; checkCIF report

## Figures and Tables

**Table 1:** Selected bond lengths (Å)

Sn1—O21	2.1001 (14)
Sn1—O11	2.1002 (14)
Sn1—C11	2.175 (2)
Sn1—C21	2.176 (2)
O11—C15	1.304 (3)
O12—C15	1.235 (3)
O21—C25	1.304 (3)
O22—C25	1.239 (3)

**Table 2:** Hydrogen-bond geometry (Å, °)

*D*—H⋯*A*	*D*—H	H⋯*A*	*D*⋯*A*	*D*—H⋯*A*
C12—H12*C*⋯O21^i^	0.98	2.54	3.362 (3)	142
C22—H22*C*⋯O11^i^	0.98	2.65	3.584 (3)	160
C16—H16*C*⋯O22^ii^	0.98	2.69	3.636 (3)	163
C16—H16*A*⋯O22^iii^	0.98	2.60	3.518 (3)	155

Symmetry codes: (i) 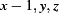; (ii) 
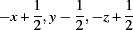
; (iii) 
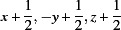
.

**Fig. 1.**
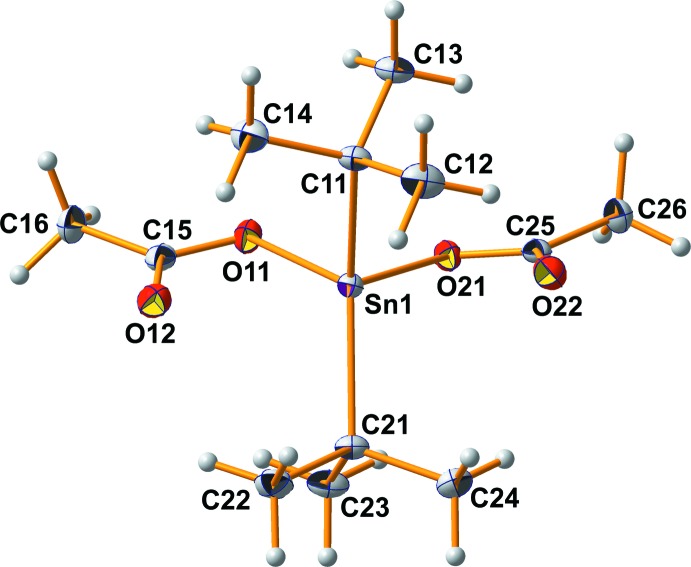
Molecular structure of the title compound, with 50% probability level displacement ellipsoids for non-H atoms.

**Fig. 2.**
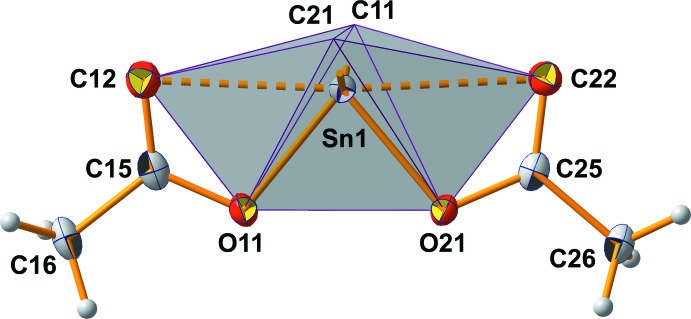
Polyhedron model of the coordination sphere of the tin atom; t-butyl groups have been omitted for clarity, weak Sn··· O interactions are indicated by dashed sticks. Displacement ellipsoids for non-H atoms are shown with 50% probability level

**Fig. 3.**
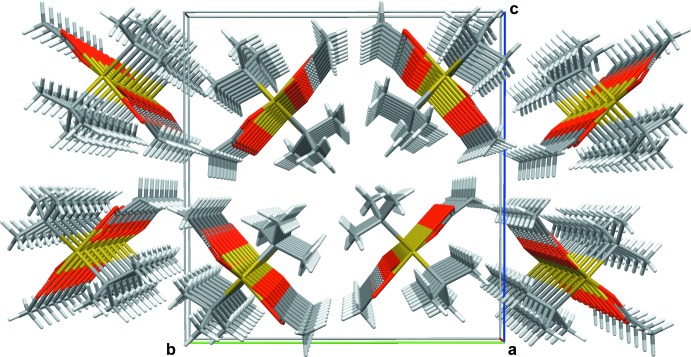
Perspective view of the crystal structure parallel to the crystallographic a axis, showing the chain-like arrangement of acetate groups and tin atoms.

**Fig. 4.**
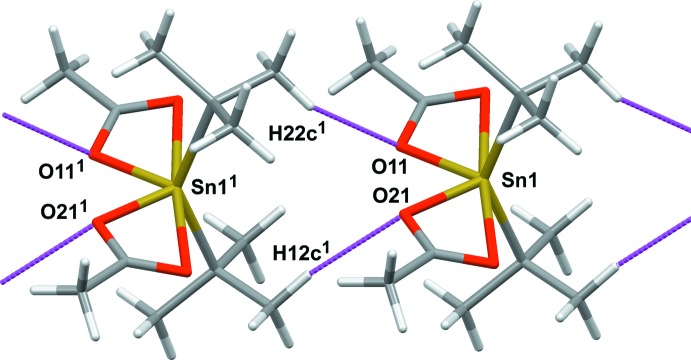
Intermolecular oxygen hydrogen interactions within the chain-like arrangement of the diacetate molecules [Symmetry operator: (1) 1 + x, y, z].

## supplementary crystallographic information

### Comment

Diorganotin(IV) dicarboxylates, *R*_2_Sn(O_2_CR')_2_, belong to the class of diorganotin compounds, *R*_2_Sn*X*_2_, with univalent anions *X*, such as the halides (*X* = F, Cl, Br, I) or alkoxides (*X* = OR'). In contrast to these anions, that behave as unidentate substitutents, the carboxylate groups, O_2_CR', can act *via* its two oxygen atoms as a bidentate ligand in a more or less symmetrical or unsymmetrical coordination mode giving rise to additional intra- or intermolecular interactions (Tiekink, 1991). In the case of diacetates, *R*' = Me, this was formerly shown for *R* = phenyl (Alcock *et al.* 1992) and *R* = methyl (Lockhart *et al.* 1987, α-modification; Mistry *et al.* 1995, β-modification) both revealing in solid state the same molecular structure type with a strongly unsymmetrical bonding mode of the acetate groups. On this background it was interesting to see whether the larger *t*-butyl groups are compatible with that structure type or not.

The asymmetric unit of the title compound (Fig. 1) consists of one formula unit with all atoms in general positions although the molecule displays a pseudo twofold rotation axis [midway C11/C21 - Sn - midway O11/O21]. In a first approximation, the tin atom is fourfold coordinated by the two *t*-butyl groups and an oxygen atom of each acetate group. Both Sn—C bond lengths are almost equal as are both Sn—O bond lengths, too (see Table 1). The last ones are comparable with Sn—O bond lengths in the other diacetates [*d*(Sn—O) = 2.076 (4), 2.079 (4), *R* = Ph; 2 *x* 2.106 (2), *R* = α-Me, 2.098 (2), 2.094 (2), *R* = β-Me]. In comparison to the methyl compounds with Sn—C bond lengths of 2 *x* 2.098 (3) Å [α], respectively 2.096 (3), 2.088 (3) Å [β] and the phenyl compound [2.119 (5), 2.110 (6) Å] Sn—C bonds of the *tert*-butyl groups are considerable longer. From the bond angles of 138.97 (7)° between the *t*-butyl groups and 79.93 (6)° between the two oxygen atoms, the coordination polyhedron is compressed to a tetragonal disphenoid (Fig. 2). Obviously, this distortion is typical for diacetates [C—Sn—C = 131.4°, *R* = Ph; 135.9 (2)°(1)°, *R* = α-Me; 133.8 (2)°, *R* = β-Me; O—Sn—O = 82.0 (3)°, *R* = Ph; 79.5 (1)°, *R* = α-Me; 80.1 (1)°, *R* = β-Me].

The coordination sphere of the tin atom is completed by the other two oxygen atoms of the acetate groups that undergo a much more weaker interaction with the tin atom [*d*(Sn···O) = 2.689 (1) Å to O22, 2.647 (2) Å to O12], resulting in a very unsymmetrical bidentate coordination mode of the acetate groups (Fig. 2). This is also reflected in two different C—O distances for each acetate group (Table 1). Again, within the molecular structures of the other diacetates a similar coordination behaviour is observed but Sn···O interactions are considerably stronger [2.583 (4), 2.527 (5) Å, *R* = Ph; 2 *x* 2.539 (2) Å, *R* = α-Me; 2.563 (2), 2.595 (2) Å, *R* = β-Me].

Both *t*-butyl groups are well ordered with a mean value for the C—C bonds of 1.527 (3) Å [range: 1.522 (3) - 1.530 (3) Å] and a mean C—C—C bond angle of 110.2 (4)° [range: 109.65 (3)° - 110.65 (3)°]. With respect to Sn—C—C bond angles, both *t*-butyl groups show similar effects: two angles are around the ideal tetrahedral value of 109.5°, whereas the third one is significantly smaller [107.09 (3)° for C12; 106.07 (2)° for C22].

In the solid, molecules are arranged in chains with the tin atoms and acetate groups defining the propagation plane (Fig. 3). This arrangement is also characteristic for both modifications of the methyl compounds but not for the phenyl one. In the present case, the intermolecular O···Sn distances of 4.692 (1) Å [O21···Sn1^1^] and 4.694 (1) Å [O11···Sn1^1^], however, are so long that seems impossible that these interactions are responsible for the supra-molecular architecture. There are, however, O···HC interactions between the acetate groups and *t*-butyl groups of neighbouring molecules (Fig. 4) that are much more attractive (Table 2). Similar interactions are found in all other diorganotin(IV) diacetates and even in the phenyl compound.

### Experimental

*Synthesis*:

0.51 g (0.68 mmol) di-*t*-butyltin oxide are dissolved in 0.25 g (4.16 mmol) of acetic acid (Fluka). The solution is stirred for 6 h at ambient temperature before the solvent is allowed to evaporate slowly. After about 1 week colourless block-like crystals are grown.

*Spectroscopic studies*:

Elemental analysis calcd (%) for C_5_H_4_O_3_: C, 41.06; H, 6.89. Found: C, 40.99; H, 6.53. ^1^H-NMR (CDCl_3_, p.p.m.): 2.09 (s,OAc, 3H), 1.35 (s, tBu, 9H, ^3^J(^1^H-^119/117^Sn = 119.1/114.1 Hz). {^1^H}-^13^C-NMR (CDCl_3_, p.p.m.): 180.59 (O_2_CCH_3_), 45.29 (C_α_-tBu, ^1^J(^13^C-^119/117^Sn) = 515.3/492.4 Hz), 29.67 (C_β_-tBu), 20.37 (O_2_CCH_3_). IR (ATR, cm^-1^): 2853.6, 1601.0, 1438.3, 1367.9, 1323.9, 1160.4, 1053.5, 1018.2, 942.5, 807.9, 688.4, 620.1, 499.2.

*Crystallographic studies*:

A suitable single-crystal was selected under a polarization microscope and mounted on a 50 µm MicroMesh MiTeGen Micromount^TM^ using FROMBLIN Y perfluoropolyether (LVAC 16/6, Aldrich).

### Refinement

Hydrogen atoms were clearly identified in difference Fourier syntheses. Their positions were idealized and refined at calculated positions riding on the carbon atoms with C—H = 0.98 Å.

### Crystal data

**Table:**

[Sn(C_4_H_9_)_2_(C_2_H_3_O_2_)_2_]	*F*(000) = 712
*M**_r_* = 351.00	*D*_x_ = 1.562 Mg m^−^^3^
Monoclinic, *P*2_1_/*n*	Mo *K*α radiation, λ = 0.71073 Å
Hall symbol: -P 2yn	Cell parameters from 6480 reflections
*a* = 6.1039 (3) Å	θ = 2.6–25.7°
*b* = 15.3928 (7) Å	µ = 1.71 mm^−^^1^
*c* = 15.9601 (8) Å	*T* = 100 K
β = 95.462 (2)°	Block, colourless
*V* = 1492.74 (12) Å^3^	0.14 × 0.06 × 0.04 mm
*Z* = 4	

### Data collection

**Table:**

Bruker APEXII CCD diffractometer	3590 independent reflections
Radiation source: fine-focus sealed tube	2980 reflections with *I* > 2σ(*I*)
Graphite monochromator	*R*_int_ = 0.081
φ and ω scans	θ_max_ = 28.0°, θ_min_ = 1.8°
Absorption correction: multi-scan (*SADABS*; Bruker, 2009)	*h* = −8→8
*T*_min_ = 0.791, *T*_max_ = 0.939	*k* = −20→19
76636 measured reflections	*l* = −21→21

### Refinement

**Table:**

Refinement on *F*^2^	Secondary atom site location: difference Fourier map
Least-squares matrix: full	Hydrogen site location: inferred from neighbouring sites
*R*[*F*^2^ > 2σ(*F*^2^)] = 0.023	H-atom parameters constrained
*wR*(*F*^2^) = 0.046	*w* = 1/[σ^2^(*F*_o_^2^) + (0.0133*P*)^2^ + 1.0511*P*] where *P* = (*F*_o_^2^ + 2*F*_c_^2^)/3
*S* = 1.04	(Δ/σ)_max_ = 0.002
3590 reflections	Δρ_max_ = 0.59 e Å^−^^3^
167 parameters	Δρ_min_ = −0.51 e Å^−^^3^
0 restraints	Extinction correction: *SHELXL*, Fc^*^=kFc[1+0.001xFc^2^λ^3^/sin(2θ)]^-1/4^
Primary atom site location: structure-invariant direct methods	Extinction coefficient: 0.00201 (16)

### Special details

**Table:**

Geometry. All e.s.d.'s (except the e.s.d. in the dihedral angle between two l.s. planes) are estimated using the full covariance matrix. The cell e.s.d.'s are taken into account individually in the estimation of e.s.d.'s in distances, angles and torsion angles; correlations between e.s.d.'s in cell parameters are only used when they are defined by crystal symmetry. An approximate (isotropic) treatment of cell e.s.d.'s is used for estimating e.s.d.'s involving l.s. planes.
Refinement. Refinement of *F*^2^ against ALL reflections. The weighted *R*-factor *wR* and goodness of fit *S* are based on *F*^2^, conventional *R*-factors *R* are based on *F*, with *F* set to zero for negative *F*^2^. The threshold expression of *F*^2^ > σ(*F*^2^) is used only for calculating *R*-factors(gt) *etc*. and is not relevant to the choice of reflections for refinement. *R*-factors based on *F*^2^ are statistically about twice as large as those based on *F*, and *R*- factors based on ALL data will be even larger.

### Fractional atomic coordinates and isotropic or equivalent isotropic displacement parameters (Å^2^)

**Table:**

	*x*	*y*	*z*	*U*_iso_*/*U*_eq_	
Sn1	0.19140 (2)	0.295768 (9)	0.285075 (8)	0.01333 (6)	
C11	0.0432 (3)	0.20445 (14)	0.19231 (13)	0.0162 (4)	
C12	−0.1709 (3)	0.24192 (16)	0.14968 (14)	0.0257 (5)	
H12A	−0.2374	0.2001	0.1085	0.031 (2)*	
H12B	−0.1396	0.2962	0.1211	0.031 (2)*	
H12C	−0.2730	0.2534	0.1922	0.031 (2)*	
C13	0.2095 (4)	0.18975 (15)	0.12781 (13)	0.0216 (5)	
H13A	0.3485	0.1689	0.1566	0.031 (2)*	
H13B	0.2350	0.2445	0.0990	0.031 (2)*	
H13C	0.1515	0.1464	0.0865	0.031 (2)*	
C14	−0.0036 (4)	0.11881 (15)	0.23553 (14)	0.0259 (5)	
H14A	−0.1128	0.1285	0.2758	0.031 (2)*	
H14B	0.1327	0.0966	0.2654	0.031 (2)*	
H14C	−0.0609	0.0764	0.1932	0.031 (2)*	
C21	0.0894 (3)	0.38924 (14)	0.37595 (12)	0.0161 (4)	
C22	−0.0723 (4)	0.34722 (15)	0.43092 (14)	0.0245 (5)	
H22A	−0.1180	0.3900	0.4713	0.030 (2)*	
H22B	−0.0011	0.2980	0.4614	0.030 (2)*	
H22C	−0.2016	0.3267	0.3953	0.030 (2)*	
C23	0.2995 (4)	0.41692 (15)	0.42919 (14)	0.0243 (5)	
H23A	0.2626	0.4591	0.4717	0.030 (2)*	
H23B	0.4014	0.4435	0.3928	0.030 (2)*	
H23C	0.3689	0.3659	0.4572	0.030 (2)*	
C24	−0.0185 (4)	0.46712 (15)	0.33009 (14)	0.0249 (5)	
H24A	−0.1502	0.4481	0.2950	0.030 (2)*	
H24B	0.0852	0.4936	0.2944	0.030 (2)*	
H24C	−0.0601	0.5098	0.3713	0.030 (2)*	
O11	0.4677 (2)	0.22581 (9)	0.33641 (9)	0.0198 (3)	
O12	0.1995 (3)	0.18113 (10)	0.40892 (10)	0.0251 (4)	
C15	0.3940 (4)	0.17649 (14)	0.39399 (13)	0.0203 (5)	
C16	0.5546 (4)	0.11407 (16)	0.43777 (14)	0.0265 (5)	
H16A	0.5282	0.1099	0.4973	0.037 (4)*	
H16B	0.7049	0.1347	0.4333	0.037 (4)*	
H16C	0.5357	0.0567	0.4114	0.037 (4)*	
O21	0.4416 (2)	0.36282 (9)	0.23048 (9)	0.0178 (3)	
O22	0.1404 (2)	0.41038 (10)	0.15759 (9)	0.0231 (3)	
C25	0.3440 (4)	0.40733 (13)	0.16819 (13)	0.0185 (5)	
C26	0.4899 (4)	0.45081 (15)	0.11080 (14)	0.0240 (5)	
H26A	0.5160	0.4114	0.0646	0.038 (4)*	
H26B	0.6306	0.4656	0.1423	0.038 (4)*	
H26C	0.4188	0.5039	0.0879	0.038 (4)*	

### Atomic displacement parameters (Å^2^)

**Table:**

	*U*^11^	*U*^22^	*U*^33^	*U*^12^	*U*^13^	*U*^23^
Sn1	0.01484 (8)	0.01307 (8)	0.01231 (8)	0.00027 (6)	0.00240 (5)	−0.00084 (6)
C11	0.0158 (10)	0.0177 (11)	0.0156 (10)	−0.0010 (8)	0.0038 (8)	−0.0048 (8)
C12	0.0162 (11)	0.0342 (14)	0.0260 (12)	0.0018 (10)	−0.0017 (9)	−0.0072 (10)
C13	0.0222 (11)	0.0235 (13)	0.0194 (11)	−0.0006 (9)	0.0037 (9)	−0.0078 (9)
C14	0.0294 (13)	0.0220 (12)	0.0276 (12)	−0.0095 (10)	0.0089 (10)	−0.0041 (10)
C21	0.0153 (10)	0.0166 (11)	0.0167 (10)	0.0006 (8)	0.0028 (8)	−0.0051 (8)
C22	0.0249 (12)	0.0239 (13)	0.0265 (12)	0.0005 (10)	0.0128 (10)	−0.0036 (10)
C23	0.0246 (12)	0.0266 (13)	0.0216 (12)	−0.0006 (10)	0.0018 (9)	−0.0093 (9)
C24	0.0303 (13)	0.0204 (12)	0.0246 (12)	0.0065 (10)	0.0057 (10)	−0.0020 (9)
O11	0.0211 (8)	0.0180 (8)	0.0199 (8)	0.0020 (6)	−0.0005 (6)	0.0035 (6)
O12	0.0273 (9)	0.0222 (9)	0.0263 (9)	0.0039 (7)	0.0055 (7)	0.0017 (7)
C15	0.0286 (13)	0.0147 (11)	0.0166 (11)	0.0014 (9)	−0.0025 (9)	−0.0031 (8)
C16	0.0337 (13)	0.0229 (12)	0.0221 (12)	0.0072 (10)	−0.0024 (10)	0.0049 (10)
O21	0.0192 (8)	0.0172 (8)	0.0174 (7)	−0.0003 (6)	0.0046 (6)	0.0027 (6)
O22	0.0226 (9)	0.0267 (9)	0.0202 (8)	0.0019 (7)	0.0028 (6)	0.0013 (6)
C25	0.0279 (12)	0.0137 (11)	0.0147 (10)	−0.0021 (9)	0.0055 (9)	−0.0036 (8)
C26	0.0304 (13)	0.0215 (13)	0.0209 (12)	−0.0012 (10)	0.0076 (10)	0.0030 (9)

### Geometric parameters (Å, º)

**Table:**

Sn1—O21	2.1001 (14)	C22—H22B	0.9800
Sn1—O11	2.1002 (14)	C22—H22C	0.9800
Sn1—C11	2.175 (2)	C23—H23A	0.9800
Sn1—C21	2.176 (2)	C23—H23B	0.9800
C11—C14	1.527 (3)	C23—H23C	0.9800
C11—C12	1.528 (3)	C24—H24A	0.9800
C11—C13	1.530 (3)	C24—H24B	0.9800
C12—H12A	0.9800	C24—H24C	0.9800
C12—H12B	0.9800	O11—C15	1.304 (3)
C12—H12C	0.9800	O12—C15	1.235 (3)
C13—H13A	0.9800	C15—C16	1.497 (3)
C13—H13B	0.9800	C16—H16A	0.9800
C13—H13C	0.9800	C16—H16B	0.9800
C14—H14A	0.9800	C16—H16C	0.9800
C14—H14B	0.9800	O21—C25	1.304 (3)
C14—H14C	0.9800	O22—C25	1.239 (3)
C21—C24	1.522 (3)	C25—C26	1.495 (3)
C21—C22	1.525 (3)	C26—H26A	0.9800
C21—C23	1.530 (3)	C26—H26B	0.9800
C22—H22A	0.9800	C26—H26C	0.9800
			
O21—Sn1—O11	79.93 (6)	C21—C22—H22B	109.5
O21—Sn1—C11	107.88 (7)	H22A—C22—H22B	109.5
O11—Sn1—C11	101.58 (7)	C21—C22—H22C	109.5
O21—Sn1—C21	102.54 (7)	H22A—C22—H22C	109.5
O11—Sn1—C21	110.43 (7)	H22B—C22—H22C	109.5
C11—Sn1—C21	138.97 (7)	C21—C23—H23A	109.5
C14—C11—C12	109.77 (18)	C21—C23—H23B	109.5
C14—C11—C13	109.93 (18)	H23A—C23—H23B	109.5
C12—C11—C13	110.51 (17)	C21—C23—H23C	109.5
C14—C11—Sn1	109.51 (14)	H23A—C23—H23C	109.5
C12—C11—Sn1	109.99 (14)	H23B—C23—H23C	109.5
C13—C11—Sn1	107.09 (13)	C21—C24—H24A	109.5
C11—C12—H12A	109.5	C21—C24—H24B	109.5
C11—C12—H12B	109.5	H24A—C24—H24B	109.5
H12A—C12—H12B	109.5	C21—C24—H24C	109.5
C11—C12—H12C	109.5	H24A—C24—H24C	109.5
H12A—C12—H12C	109.5	H24B—C24—H24C	109.5
H12B—C12—H12C	109.5	C15—O11—Sn1	104.81 (13)
C11—C13—H13A	109.5	O12—C15—O11	120.3 (2)
C11—C13—H13B	109.5	O12—C15—C16	123.1 (2)
H13A—C13—H13B	109.5	O11—C15—C16	116.6 (2)
C11—C13—H13C	109.5	C15—C16—H16A	109.5
H13A—C13—H13C	109.5	C15—C16—H16B	109.5
H13B—C13—H13C	109.5	H16A—C16—H16B	109.5
C11—C14—H14A	109.5	C15—C16—H16C	109.5
C11—C14—H14B	109.5	H16A—C16—H16C	109.5
H14A—C14—H14B	109.5	H16B—C16—H16C	109.5
C11—C14—H14C	109.5	C25—O21—Sn1	106.07 (13)
H14A—C14—H14C	109.5	O22—C25—O21	120.25 (19)
H14B—C14—H14C	109.5	O22—C25—C26	123.21 (19)
C24—C21—C22	109.65 (17)	O21—C25—C26	116.51 (19)
C24—C21—C23	110.46 (18)	C25—C26—H26A	109.5
C22—C21—C23	110.65 (17)	C25—C26—H26B	109.5
C24—C21—Sn1	109.79 (13)	H26A—C26—H26B	109.5
C22—C21—Sn1	110.17 (14)	C25—C26—H26C	109.5
C23—C21—Sn1	106.07 (13)	H26A—C26—H26C	109.5
C21—C22—H22A	109.5	H26B—C26—H26C	109.5

### Hydrogen-bond geometry (Å, º)

**Table:**

*D*—H···*A*	*D*—H	H···*A*	*D*···*A*	*D*—H···*A*
C12—H12*C*···O21^i^	0.98	2.54	3.362 (3)	142
C22—H22*C*···O11^i^	0.98	2.65	3.584 (3)	160
C16—H16*C*···O22^ii^	0.98	2.69	3.636 (3)	163
C16—H16*A*···O22^iii^	0.98	2.60	3.518 (3)	155

Symmetry codes: (i) *x*−1, *y*, *z*; (ii) −*x*+1/2, *y*−1/2, −*z*+1/2; (iii) *x*+1/2, −*y*+1/2, *z*+1/2.
